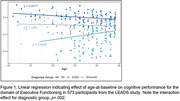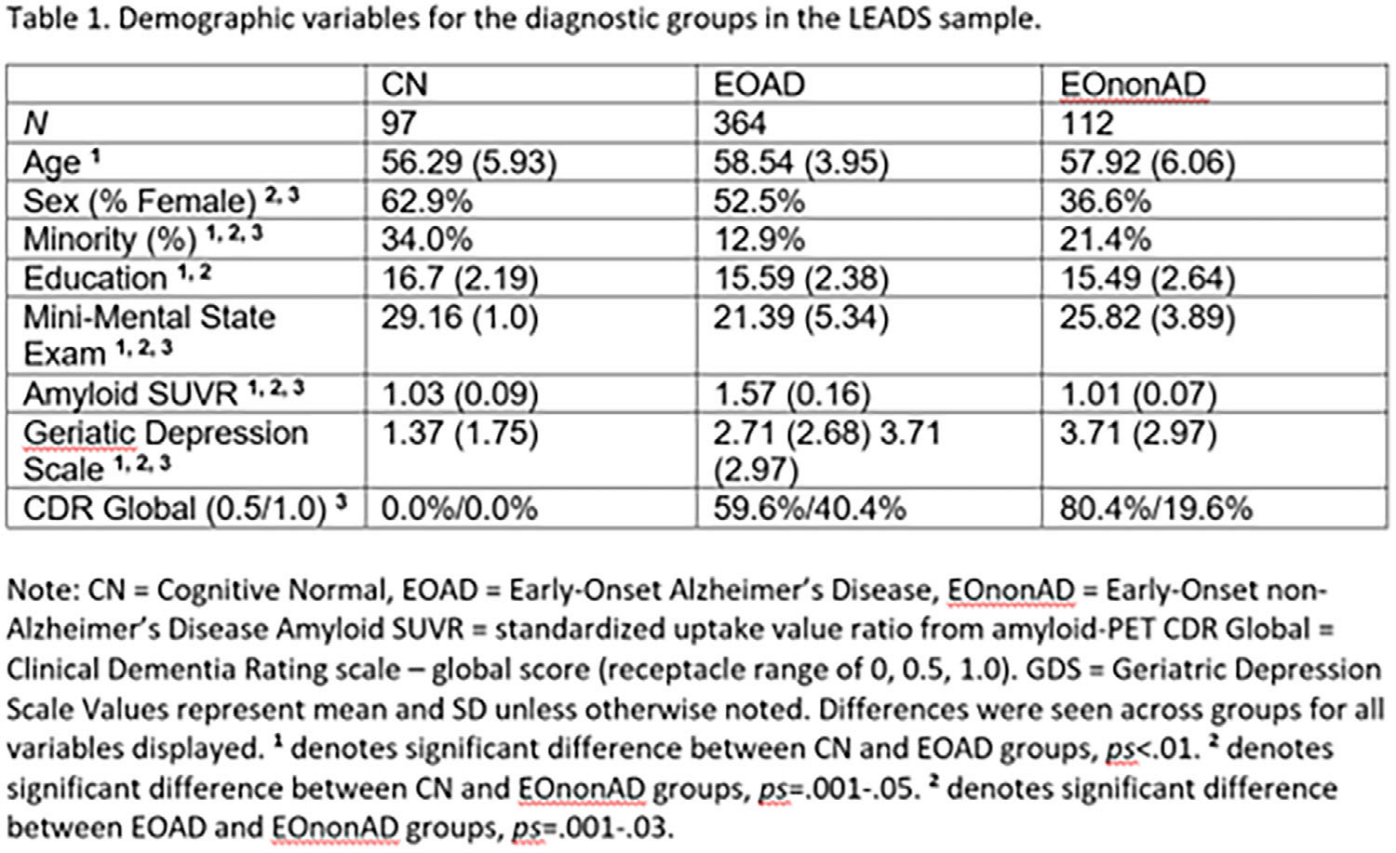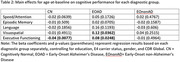# Association Between Age and Cognitive Severity in Early‐Onset AD: Extension of preliminary findings in the Longitudinal Early‐Onset Alzheimer’s Disease Study (LEADS)

**DOI:** 10.1002/alz.092663

**Published:** 2025-01-03

**Authors:** Dustin B. Hammers, Ani Eloyan, Alexander Taurone, Maryanne Thangarajah, Kala Kirby, Bonnie Wong, Jeffrey L. Dage, Kelly N. Nudelman, Maria C. Carrillo, Gil D. Rabinovici, Bradford C. Dickerson, Liana G. Apostolova, LEADS Consortium

**Affiliations:** ^1^ Indiana University School of Medicine, Indianapolis, IN USA; ^2^ Department of Biostatistics, Brown University, Providence, RI USA; ^3^ Brown University, Providence, RI USA; ^4^ Department of Neurology, Indiana University School of Medicine, Indianapolis, IN USA; ^5^ Frontotemporal Disorders Unit and Massachusetts Alzheimer’s Disease Research Center, Department of Neurology, Massachusetts General Hospital and Harvard Medical School, Boston, MA USA; ^6^ Department of Medical and Molecular Genetics, Indiana University School of Medicine, Indianapolis, IN USA; ^7^ Alzheimer’s Association, Chicago, IL USA; ^8^ Memory and Aging Center, Weill Institute for Neurosciences, University of California, San Francisco, San Francisco, CA USA; ^9^ Department of Radiology and Imaging Sciences, Indiana University School of Medicine, Indianapolis, IN USA

## Abstract

**Background:**

Widespread cognitive impairments have previously been documented in Early‐Onset Alzheimer’s Disease (EOAD) relative to cognitively normal (CN) same‐aged peers or those with cognitive impairment without amyloid pathology (Early‐Onset non‐Alzheimer’s Disease; EOnonAD; Hammers et al., 2023). Prior preliminary work has similarly observed worse cognitive performance being associated with earlier ages in EOAD participants enrolled in the Longitudinal Early‐Onset Alzheimer’s Disease Study (LEADS; Apostolova et al., 2019). It is unclear, however, if these age effects are seen across early‐onset conditions, and whether cognitive discrepancies among diagnostic groups are uniform across the age spectrum. The objective of the current study is to more‐extensively examine the impact of age‐at‐baseline on cognition within LEADS, with emphasis placed on the influence of diagnostic group on these associations.

**Method:**

Expanded cross‐sectional baseline cognitive data from 573 participants (CN, *n* = 97; EOAD, *n* = 364; EOnonAD, *n* = 112) enrolled in the LEADS study (aged 40‐64) were analyzed. Multiple linear regression analyses were conducted to investigate associations between age‐at‐baseline and cognition for each diagnostic group – and their interaction among diagnoses – controlling for gender, education, *APOE* ε4 status, and disease severity.

**Result:**

See **Table 1** for demographic characteristics of our sample. Linear regression showed a significant interaction effect for the cognitive domain of Executive Functioning (*p* = .002). Specifically, while the EOAD group displayed a positive relationship between age‐at‐baseline and Executive Functioning performance (*β* = 0.08, *p* = .02; **Figure 1**), the CN group displayed a negative relationship (*β* = ‐0.04, *p* = .008) and the EOnonAD group displayed no relationship (*β* = ‐0.01, *p* = .50). A similar main‐effect for age was observed for the EOAD group when examining Visuospatial Skills (*β* = 0.12, *p* = .04), however no other age effects were evident across other diagnostic groups or cognitive domains (Episodic Memory, Language, or Speed/Attention; **Table 2**).

**Conclusion:**

Building off preliminary work, our results suggest that executive functioning may be disproportionately impacted earlier in the disease course in participants with EOAD relative to other diagnostic groups. This finding appears to be unique to executive functioning, as it was absent in other cognitive domains and remained after accounting for disease severity. This highlights the need for further investigation into executive dysfunction early in the course of EOAD.